# Direct Tensile Strength and Characteristics of Dentin Restored with All-Ceramic, Resin-Composite, and Cast Metal Prostheses Cemented with Resin Adhesives

**DOI:** 10.1155/2015/656948

**Published:** 2015-10-11

**Authors:** Morakot Piemjai, Nobuo Nakabayashi

**Affiliations:** Department of Prosthodontics, Faculty of Dentistry, Chulalongkorn University, Henri-Dunant Road, Pathumwan, Bangkok 10330, Thailand

## Abstract

A dentin-cement-prosthesis complex restored with either all-porcelain, cured resin-composite, or cast base metal alloy and cemented with either of the different resin cements was trimmed into a mini-dumbbell shape for tensile testing. The fractured surfaces and characterization of the dentin-cement interface of bonded specimens were investigated using a Scanning Electron Microscope. A significantly higher tensile strength of all-porcelain (12.5 ± 2.2 MPa) than that of cast metal (9.2 ± 3.5 MPa) restorations was revealed with cohesive failure in the cement and failure at the prosthesis-cement interface in Super-Bond C&B group. No significant difference in tensile strength was found among the types of restorations using the other three cements with adhesive failure on the dentin side and cohesive failure in the cured resin. SEM micrographs demonstrated the consistent hybridized dentin in Super-Bond C&B specimens that could resist degradation when immersed in hydrochloric acid followed by NaOCl solutions whereas a detached and degraded interfacial layer was found for the other cements. The results suggest that when complete hybridization of resin into dentin occurs tensile strength at the dentin-cement is higher than at the cement-prosthesis interfaces. The impermeable hybridized dentin can protect the underlying dentin and pulp from acid demineralization, even if detachment of the prosthesis has occurred.

## 1. Introduction

Dentin is more susceptible to degradation by acid and wear when exposed to the acidic oral cavity compared with enamel due to the smaller crystal size of hydroxyapatite, the presence of tubules, and its greater organic content. This natural phenomenon has not been well recognized in our community. Good retention has been believed to be one of the major requirements in achieving long-term success of restorations and fixed prostheses, as the explanation for the detachment of restorations was due to poor retention. Therefore researchers have attempted to increase retention either by removal of sound tooth substance to create mechanical interlocking for abutments or by increasing the strength of the cement bond to both tooth and restorations [1–4]. However the most common failure found in either direct or indirect restorations and fixed partial dentures is secondary caries [4–8], especially located at the gingival margins [6].

Adhesive resin cements have been increasingly used as they provided better retention than acid-base cements [9–11]. Mostly the retention provided by acid-base cements has been evaluated in terms of crown retention by using the pulling force required to remove the crown [11–13]. With this technique it is difficult to control the size of the interfacial area and the stress distribution, mainly shearing stress, through the tooth-cement-prosthesis junctions. The method recommended to measure the bond strength to tooth substrates in the ISO/TC 106 Dentistry Standard is not suitable to identify defects created in the subsurface dentin, as the cross-sectional area of the tooth substrate is wider than that of the bonded interface [10, 14]. Tensile strength measurement using dumbbell specimens is a widely accepted methodology in materials science and engineering to detect the defect in materials [15]. A mini-dumbbell shaped specimen (3 × 2 × 1.2 mm rod forming the resin-dentin junction) modified to obtain reliable data for the full dentin depth was proposed to find defects or the weakest part in the restored dentin [10, 16–19]. A reliable tensile strength of resin bonded to dentin depends on the quality of the hybridized dentin or hybrid layer [10]. It has been reported that specimens with initial high tensile bond strength as measured using a microtensile test degraded after 1–3 years [20, 21]. Tensile testing using mini-dumbbell specimens and a chemical challenge using hydrochloric acid (HCl) and sodium hypochlorite (NaOCl) solutions have been shown to be effective in detecting defects such as demineralized dentin and smears in the restored dentin [16–19]. Adhesive failure and cohesive failure in the remaining demineralized dentin suggest that the resin infiltration into the conditioned dentin was not complete. The dual immersion in HCl and NaOCl solutions removes the mineral phase and the collagen fibers that have not been enveloped by impregnated resin. Degradation of dentin-resin interfacial layer after chemical challenge confirms the existence of demineralized dentin. These defects can lead to leakage under restorations bonded to dentin [22, 23]. Demineralized dentin permeable to dyes, whether formed by acid-base cements before setting or formed by incomplete infiltration of resin [22, 23], permits diffusion of acid which subsequently may result in tooth hypersensitivity, short-term detachment of restorations, secondary caries, and pulpal pathology [4–8, 24–26]. Microleakage-free restorations can be achieved on restored dentin when complete hybridization of dentin occurs, because an impermeable hybridized dentin layer is formed [22, 23, 27].

Although bonding resin to enamel and dentin protected by a barrier impermeable to acids may maintain the retention for restorations, preparation of impermeable hybridized dentin is not as simple as for enamel as dentin contains more organic substances and water which can interfere with the diffusion of monomers [17, 20, 21]. Numerous adhesive resin systems, both for direct bonding and for cementation, have been developed and released into the market over the last 40 years. The adverse effect of phosphoric acid in removing a weak smear layer must be carefully studied in order to understand dentin substrates suitable for clinically reliable bonding. The acid dissolves water soluble hydrophilic glycosaminoglycans (GAGs), immobilized in intact dentin with hydroxyapatite, into the demineralized dentin. This must be the reason why demineralized dentin is so hydrophilic and difficult to dehydrate [28–30].

Many methods have been introduced to ensure sufficient tensile strength between resin cements and prosthetic materials. These include preparations for mechanical retention by grinding with burs, air abrasion with aluminum oxide, and/or etching with either acidic solutions or electrolysis [31–33]. The use of silane coupling agents was reported to increase the bond strength of resin to porcelain as well as to cured resin-composite, as it promoted chemical adhesion [33–35]. Surface treatment using an alloy primer has also been reported to significantly increase the tensile bond strength both for base metal and noble alloys [36].

The authors hypothesized that direct tensile strength of dentin-cement-prosthesis restoration using mini-dumbbell shaped specimens [10, 16–19] and the characteristics of the dentin-cement interface may indicate that the high strength of the restorative materials is not as important as the protection of exposed basic dentin with an impermeable barrier resistant to acid demineralization for long-term function. In other words protecting exposed dentin with a barrier impermeable to acid for demineralization is critical for the long-term success of restored dentin with either brittle tooth-colored materials or stronger cast metal.

The objective of this study was to detect the weakest area in the restored dentin-cement-prosthesis complex when restored with cast metal, cured resin-composite, and all-porcelain cemented to dentin with different resin cements, using a direct tensile strength test and the characterization of dentin-cement interface.

## 2. Materials and Methods

### 2.1. Part I: Direct Tensile Strength Test

#### 2.1.1. Preparation of Dentin Slabs

**Figure 1:**
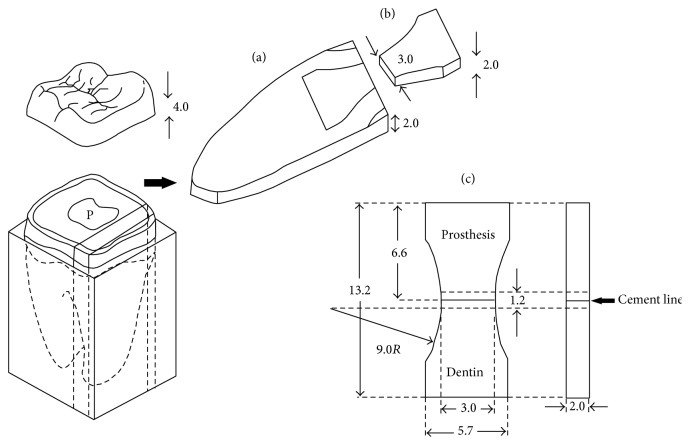
Schematics (in mm) of a dentin slab (a), restored with half mini-dumbbell prosthesis (b), to prepare a mini-dumbbell specimen (c). P: pulp chamber.

Extracted human molars that had been removed and frozen for less than three months were root-embedded in acrylic blocks (Taklon Co., Milan, Italy). The teeth required extraction and the patients gave written informed consent for their use in this project. A 4.0 mm occlusal portion was cut off horizontally and vertically sectioned to prepare 2.0 mm dentin slabs (Figure 1) using a sectioning machine (Isomet, Struers Co., Copenhagen, Denmark). Twelve dentin slabs for each group were cut. The cross-sectioned surfaces of the slabs were prepared using a diamond cylinder bur (GC International Co., Aichi, Japan) for cementing with the prostheses. A mini-dumbbell template was used to outline the bonding interface area on each dentin slab for tensile testing (Figure 1(a)).

#### 2.1.2. Fabrication of Prostheses

A standardized mini-dumbbell plastic mold was prepared. Mini-dumbbell patterns using self-cured acrylic resin (Taklon Co., Milan, Italy) with a 2.0 × 3.0 mm cross-section on the center [17] were prepared using the standardized mold. Acrylic resin patterns were sprued and invested using PowerCast investment (Whip Mix Co., Kentucky, USA) with a powder/liquid ratio of 100 g/23 mL. The pattern mold was cast into base metal alloys (Ni-Cr alloy, Wiron 99, Bego Co., Bremen, Germany) using an electronic induction casting machine (Degutron, Degussa, Germany) at the casting temperature of approximately 1450°C. Each casting was quenched, divested, and finished with stones and rubber finishing burs. A carborundum disc (Jota Co., Ruthi, Switzerland) was used to cut through the center of the mini-dumbbell to prepare two half mini-dumbbells (Figure 1(b)).

Resin-composite (Filtek Z 250, 3M ESPE, St. Paul, MN, USA) mini-dumbbell specimens were prepared using the standardized plastic mold. The mold was filled with resin-composite with a bulk placement and light-cured for 60 s on each side using a light curing machine (3M Elipar Trilight, St. Paul, MN, USA). Standardized mini-dumbbell investment molds were prepared to make all-porcelain specimens using the dentin powder (Vita Omega, VITA Zahnfabrik, Bad Säckingen, Germany). The firing cycle of porcelain furnace (Vita Vacumat, Bad Säckingen, Germany) recommended by a manufacturer was scheduled (Table 1). Three layers of porcelain build-up were applied into the mold. After being finished with the stones and rubber finishing burs all resin-composite and porcelain mini-dumbbell specimens were sectioned with diamond discs (Jota Co., Ruthi, Switzerland) to prepare two half mini-dumbbells for each specimen (Figure 1(b)).

#### 2.1.3. Mini-Dumbbell Preparation of Restored Dentin

All the twelve half mini-dumbbell shape prostheses for each group were air-blasted with 50 *µ*m alumina for 10 s on the surface areas to be cemented. A silane coupling agent specific for each cement system was applied on resin-composite and porcelain surfaces before fixing on dentin slabs with resin cements. The bonding procedures followed the manufacturer's recommendation as shown in Table 2. The same operator cemented all the restorations using finger force. After light curing or initial autocuring of resin cements, each bonded sample was trimmed into a mini-dumbbell shaped specimen with the cross-section of 2.0 × 3.0 mm and 1.2 mm rod high (Figure 1(c)), using a diamond cylinder bur and high speed handpiece (KaVo Dental Co., NC, USA) with air-water spray.

#### 2.1.4. Preparation for Tensile Testing

**Figure 2:**
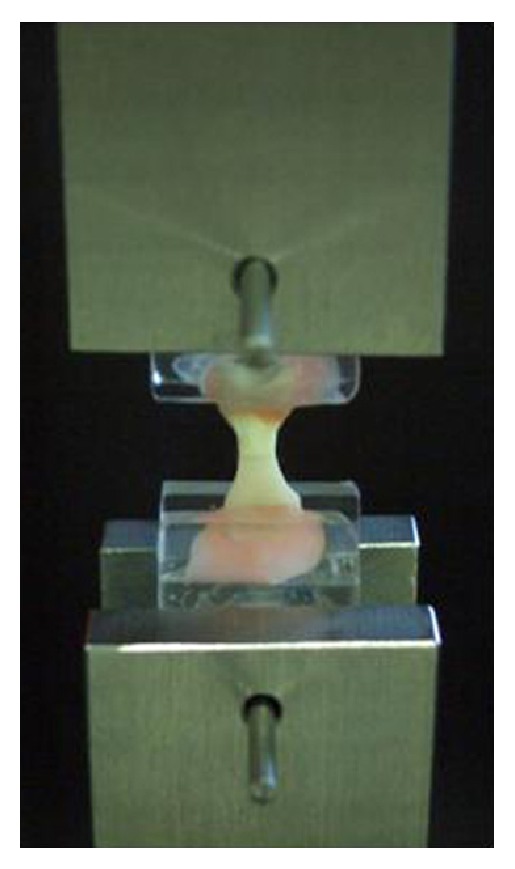
Direct tensile testing of restored dentin with porcelain using a universal testing machine.

After storing in water at 37°C for 24 h, all mini-dumbbell bonded specimens in all groups were affixed to the poly(methyl methacrylate) (PMMA) jigs using Super-Bond C&B (Sun Medical, Shiga, Japan) and self-cured acrylic resin for tensile testing. With a cross-head speed of 1.0 mm/min, a tensile force was applied using a universal testing machine (Lloyd Co., Hampshire, UK) on the assembled specimen (Figure 2). The cross-sectional areas of fractured specimens were remeasured using a digital micrometer (Mitutoyo 293, Tokyo, Japan). The tensile strength data were calculated in MPa and statistically analyzed using an analysis of variance (ANOVA) and Scheffe's test. Fracture surfaces of specimens in each group were investigated using a light microscope (Nikon, Tokyo, Japan) and a Scanning Electron Microscope (SEM, JSM-5008LV, JEOL, Tokyo, Japan) to categorize the mode of failures.

### 2.2. Part II: Characterization of the Dentin-Cement Interfacial Layer

Three dentin slabs similarly prepared as mentioned in part I for each cement group were restored with three veneers of light-cured resin-composite (2 × 4 × 1 mm) using each resin cement to characterize the dentin-cement interface. The manipulation procedures followed the manufacturer's recommendation as previously described (Table 2). Without epoxy embedding, two cross-sectional specimens of 1 mm thickness were prepared from each restored dentin specimen using a diamond disc and low-speed handpiece. The prepared surface was abraded on 600-grit and then 1,200-grit abrasive papers and then polished with 0.05 *µ*m alumina paste. Specimens were ultrasonically cleaned for 30 min and air-dried. One was immersed in 6 mol/L HCl for 30 s followed by 1% NaOCl for 60 min. All the polished and chemically treated specimens were desiccated and gold sputtered. The thickness of the dentin-resin interfacial layer on the chemically treated and the originally polished specimens was compared using SEM micrographs at ×500 and ×2000 magnification.

## 3. Results

**Figure 3:**
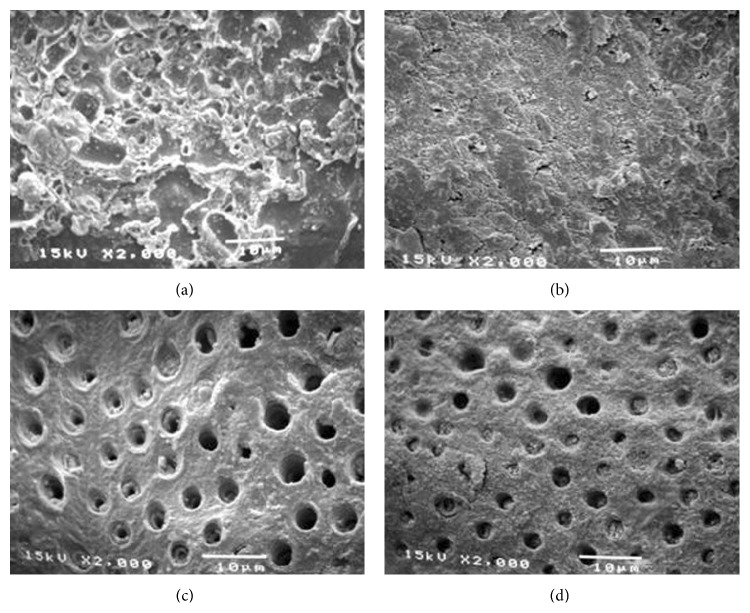
Fracture surface of restored dentin demonstrating (a) cohesive failure in resin and at prosthesis side interface in Super-Bond C&B specimen; (b) cohesive failure in hybridized smears and resin in PanaviaF specimen; (c) adhesive failure at demineralized dentin interface in Single-Bond + RelyX and Variolink II (d) specimens.

The mean tensile strength ± standard deviation (SD), failure mode, and the amount of detached specimens during trimming of each group are shown in Table 3. A two-way ANOVA found significant differences in tensile strength among the types of cements and prostheses. A Scheffe test at *P* < 0.05 revealed significant differences between groups of cement and prosthesis types (Table 3). The highest tensile strength of restored dentin was found when using Super-Bond C&B cement with a cohesive failure in the cured resin and adhesive failure on the prosthesis side interface (Figure 3(a)). No significant difference in tensile strength between PanaviaF (Kuraray Medical Inc., Okayama, Japan) and Variolink II (Ivoclar Vivadent, Liechtenstein) cements was found. The failure mode of PanaviaF specimens mostly occurred with mixed failure of adhesive on the dentin side interface and cohesive failure in the hybridized smear layer and resin (Figure 3(b)), while adhesive failure on the demineralized dentin interface was mostly found in Single-Bond + RelyX (3M Dental Products, St. Paul, USA) and Variolink II specimens (Figures 3(c) and 3(d)). Dentin restored with Single-Bond + RelyX showed the lowest tensile strength and greatest number of detached specimens during dumbbell preparation with adhesive failure on the dentin side interface (Figure 3(c)).

None of the specimens was detached while trimming and no adhesive failure on the dentin side interface was found in the Super-Bond C&B groups (Table 3). Most failures occurred on the prosthesis side interface, with significantly higher tensile strength for all-porcelain compared with those of cast metal restorations being revealed. No significant difference between types of prosthesis was found in the other three cement groups.

**Figure 4:**
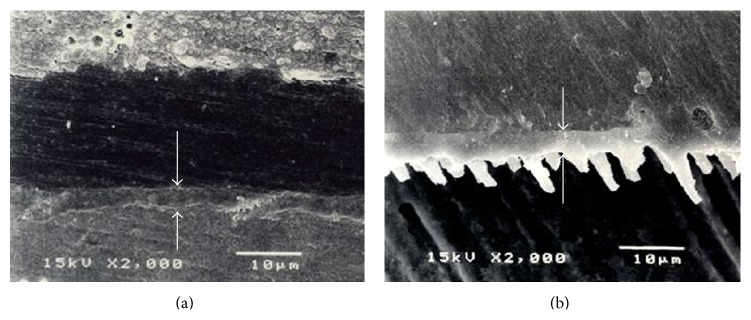
Characteristics of dentin-cement interfacial layer before (a) and after (b) HCl and NaOCl modifications demonstrated the consistent and continuous hybridized dentin (arrowed) in Super-Bond C&B specimen.

**Figure 5:**
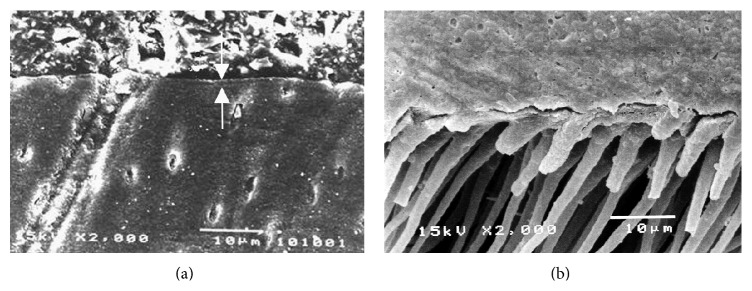
Characteristics of dentin-cement interfacial layer in PanaviaF specimen demonstrating (a) the thin layer of polished specimen (arrowed) and (b) the degraded and detached layer after HCl and NaOCl modifications.

**Figure 6:**
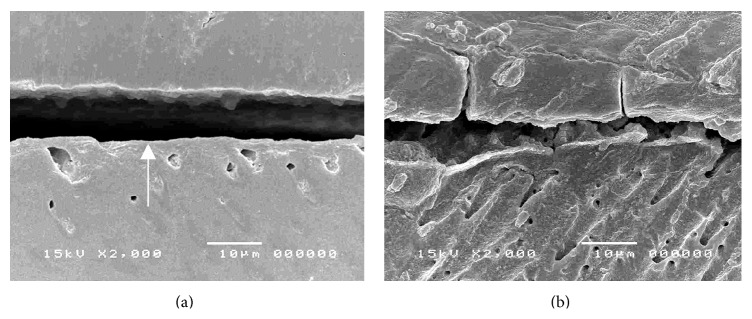
Characteristics of dentin-cement interfacial layer in Single-Bond + RelyX specimen demonstrating (a) the detachment at dentin side interface (arrowed) of polished specimen which was degraded (b) after HCl and NaOCl modifications.

**Figure 7:**
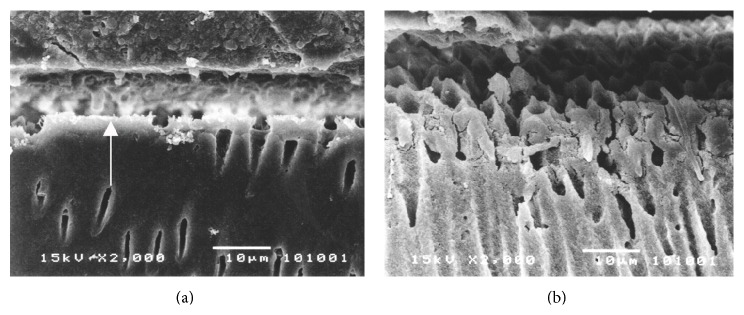
Characteristics of dentin-cement interfacial layer in Variolink II specimen demonstrating (a) the detachment at dentin side interface (arrowed) of polished specimen which was degraded (b) after HCl and NaOCl modifications.

The dentin-cement interfacial layer of Super-Bond C&B specimens was consistent and continuous for 3-4 *µ*m both before and after chemical modification using HCl and NaOCl (Figure 4), whereas that in PanaviaF specimens was detached and degraded (Figure 5). In Variolink II and Single-Bond + RelyX specimens, where dentin was demineralized by phosphoric acid and kept moist, the interfacial layer was detached on the dentin side interface and was degraded after the chemical modification (Figures 6 and 7).

## 4. Discussion

A significantly higher tensile strength of restored dentin was found for Super-Bond C&B specimens with cohesive failure in cement and failure on the prosthesis side interfaces (Figure 3(a)). The hybridized dentin before and after chemical immersion was consistent and continually attached (Figure 4). These results suggested that dentin conditioned with 10% citric acid and 3% ferric chloride (10-3) solution, rinsed, and gently air-dried could provide permeability for complete infiltration of 4-methacryloyloxyethyl trimellitate anhydride in methyl methacrylate initiated by tri-*n*-butyl borane (4-META/MMA-TBB) in the presence of PMMA resin to form an impermeable hybridized dentin layer which could resist the acid and NaOCl challenge (akin to caries formation). This means that the hydroxyapatite was well encapsulated and protected with impregnated impermeable copolymers against acid demineralization and exposed collagen was also well enveloped and protected against NaOCl degradation. The well encapsulated hydroxyapatite crystals in the hybrid layer contribute to the longevity of bonding [37, 38]. The hybridization of dentin substrate with the resin gave a higher tensile strength than did the interface of cured cement-restorative materials irrespective of whether being cast metal, cured resin-composite, or porcelain. Nevertheless, the tensile strength was sufficient to resist stress during the mini-dumbbell shape preparation as none of these restored dentin specimens was detached prior to tensile testing.

The significantly lower tensile strength of dentin restored with PanaviaF, Variolink II, and Single-Bond + RelyX specimens resulted from adhesive failure on the dentin side of the interface. This clearly suggested that good retention to restored dentin did not depend on the strength of resin cement but was due to the complete hybridization of the resin into dentin which is the substrate [27]. Adhesive failure on the dentin interface suggested that phosphoric acid conditioned dentin rinsed and kept moist had less permeability for impregnation by monomers; thus complete hybridization of resin into the conditioned dentin did not occur. This adhesive failure must be due to the weak layer of demineralized dentin in the restored dentin. It is important to discover how to eliminate this weak layer from the restored dentin. Thus the influence of GAGs dissolved in demineralized dentin by these etching agents used to remove the weak smear layer and the effect of ferric ions to aggregate GAGs to improve bonding to dentin need further study [28–30].

Cohesive failure in hybridized smear layers was also confirmed in the PanaviaF group (Figure 3(b)) as this self-etching cement bonded through the smear layers. Observation of the dentin-cement interfacial layer using SEM showed a degraded and detached layer after chemical modification (Figure 5). These results suggested that the smear layer could reduce the amount of monomer infiltration into underlying dentin and also contribute to the weakness of hybridized smear layer [10, 39]. The failure mode of Single-Bond + RelyX specimens was mostly adhesive failure on the demineralized dentin interface as for the Variolink II specimens (Figures 3(c) and 3(d)). The dentin-cement interfacial layer of these two groups demonstrated detachment of polished specimen (Figures 6(a) and 7(a)) which was degraded after chemical challenge (Figures 6(b) and 7(b)). This confirmed that monomer infiltration was difficult and could not fill the phosphoric acid demineralized dentin [29]; therefore any exposed collagen which was not enveloped in Variolink II and Single-Bond + RelyX groups was liable to degradation with NaOCl as shown in Figures 6 and 7, respectively. Thus it must be difficult for these adhesive resins to inhibit the detachment of restorations when being under stress.

The number of detached specimens during the preparation of mini-dumbbell shape found in three cement groups (Table 3) was 8, 5, and 1 out of 36 in Single-Bond + RelyX (22%), Variolink II (14%), and PanaviaF (3%), respectively. SEM micrographs of the detached surfaces showed adhesive failure on the dentin side interface. The higher percentage of detachment of restorations was probably due to restored dentin in the presence of demineralized dentin, the weakness of the dentin itself, and not because of the weakness of dental materials. These results also suggested that these cements must be carefully applied in the clinic as any demineralized dentin introduced during treatment could later be penetrated by acid produced in the mouth. The demineralized dentin resulting from the incomplete infiltration of monomers into conditioned dentin leads to leakage [22, 40, 41], degradation [38, 40], and detachment [10, 42] at this area. Thus prostheses or restorations cemented with these resins may not provide leakage-free restorations, and tooth hypersensitivity and restoration detachment could be expected in the short term, where additional increased retention of restorations or prostheses has been gained from tooth preparation geometry, or bonding to enamel, secondary caries, or pulpal pathology could be the subsequent results. This suggests that retention-based dentistry may not be the solution for long-term function of restored teeth.

Hybridized dentin that resisted the HCl and NaOCl challenge suggested that it could protect prepared weak dentin against the demineralization with lactic acid under oral condition and thus inhibit recurrent caries formation. Dentin restored using Super-Bond C&B can provide not only a microleakage-free interface [22, 23, 27, 40, 41], but also a reliable and higher tensile strength on the dentin-resin cement interface than that of the cement-restorative material interface used in this study. Significant difference in the tensile strength of the cement-restoration interface was found between porcelain and cast metal cemented with Super-Bond C&B resin. This suggested that the roughened surface on the cast metal could provide retention to resin cements able to resist a tensile stress similar to that of cured resin-composite even with a silane application. However, the greater amount of silica coupled with silane in all-porcelain compared with cured resin-composite could create a higher resistance to tensile stress than that of cast metal. With the compressive strength higher than the bite force of posterior molars [43] all-porcelain restoration coupled with this complete hybrid layer can provide the retention, strength, and stability for long-term function of both anterior and posterior teeth.

No significant difference in tensile strength between types of restorative materials was found in the Single-Bond + RelyX, Variolink II, and PanaviaF groups (Table 3). As most failure occurred on the dentin side of the interface of these cements, this suggested that the strength of resin cements and/or prostheses and the marginal fit of restorations/prostheses had no influence on the protection of restored dentin when not coupled with a complete hybrid layer. On the contrary, a barrier impermeable to acids can protect weak exposed dentin from acid demineralization, and this must contribute to the reliability of the dental treatment. Should the restoration or prosthesis detach or fracture, the remaining tooth substance will still be protected from degradation in the oral environment and can be restored again with minimal or no further tooth reduction.

The direct tensile test of the restored dentin-cement-prosthesis complex with mini-dumbbell shape modified from dentin-cement-PMMA rod specimens [10, 16–19] to simulate clinical treatment can detect the weakest area in restored dentin. Pretest failure, adhesive failure at the dentin side interface and smears, and defects in the dentin-resin interface suggest that monomer impregnation of the resin adhesives into the conditioned dentin was not complete. The chemically resistant hybrid layer is more reliable in preventing caries related to restoration. The mini-dumbbell tensile test and the characterizing of dentin-resin interface can be the basic test method required for predicting clinical performance as it can detect any defects left in the restored dentin in 24 hours while a detachment of the resin-dentin interface was caused by gradual hydrolysis of the existing demineralized dentin appearing after soaking in water for 1–5 years [38].

## 5. Conclusion

Chemically impermeable hybridized dentin in the Super-Bond C&B group provided a higher tensile strength on the dentin-cement interface than on the cement-prosthesis interface with failure in the cured resin and on the prosthesis side of the interface. Types of restorative materials had no influence in terms of retention and dentin protection in Variolink II and Single-Bond + RelyX groups, as demineralized dentin introduced during treatment was the weakest point allowing acid penetration and chemical degradation of restored dentin. These results suggest dentin restored by providing an impermeable hybridized dentin is more significant in protecting weak dentin in a cavity or abutment from demineralization with oral acids, thus promoting longer-term function. Tensile strength of restored dentin using all-porcelain or resin-composite cemented with Super-Bond C&B was not less than that of cast metal alloy which confirms that metal-free restorations can be used as well as cast metal to provide retention, stability, and perfect seal for restored dentin abutments.

## Figures and Tables

**Table 1:** The firing cycle of all-porcelain mini-dumbbell specimens.

Porcelain	Predrying		Heating-up		End firing		Vacuum
build-up	(°C)	(min)	(min)	(°C/min)	(°C)	(min)	(min)
1st firing	600	6.00	6.00	55	930	1.00	6.00
2nd firing	600	6.00	6.00	53	920	1.00	6.00
3rd firing	600	6.00	6.00	51	910	1.00	6.00

**Table 2:** Cementing procedures.

Procedures	Super-Bond C&B	PanaviaF	Variolink II	Single-Bond + RelyX
**Primer** for all-porcelain and cured composite	Porcelain liner M Liquid A : B (1 drop : 1 drop) Mixed and applied with brush	Clearfil porcelain bond activator Applied with brush	Monobond S Applied for 60 s and gently air-dried	3 M Scotchbond ceramic primer Applied and gently air-dried

**Conditioner**	10-3	ED primer	37% phosphoric acid	32% phosphoric acid

(i) Application on dentin surface	Applied 10 s, rinsed off 10 s, and air-dried 10 s	Applied 60 s and air-dried 2-3 s	Applied 10 s, rinsed off 15 s, and air-dried 2-3 s	Applied 15 s, rinsed off 10 s, blot-dried, and kept moist

**Bonding agents/resin cements** (i) Manipulations	4-META/MMA : TBB = 4 drops : 1 drop Mixed and applied on conditioned dentin and prosthesis using brush-dip technique with PMMA powder, cemented, and self-cured	Base : catalyst (paste) = 1 : 1 Hand mixed, cemented, Oxyguard coated, and light-cured 20 s (each side)	Applied Syntac primer 15 s, gently air-dried 2-3 s, applied Syntac adhesive 10 s, gently air-dried and light-cured for 20 s, applied Heliobond on both dentin and prosthesis, gently air-dried Hand mixed base and catalyst paste (1 : 1), applied on the prosthesis, cemented, and light-cured 40 s (each side)	Applied Single-Bond, gently air-dried 2–5 s (twice) on prepared dentin (light-cured 10 s) and prosthesis Hand mixed base and catalyst paste (1 : 1), applied on the prosthesis, cemented, and light-cured 40 s (each side)

**Table 3:** Mean tensile strength ± SD, failure mode in restored dentin, and numbers of detached specimens during trimming of each group.

Groups (*n* = 12)		Mean ± SD (MPa)	Failure mode in restored dentin (numbers of specimens)	Numbers of detached specimens
Cements	Prostheses			
PanaviaF^a^	Metal	4.3 ± 1.7	A/D (2), A/D + Hs + R (7), A/P + Hs + R (2)	1
	Composite	5.7 ± 4.2	A/D (2), A/D + Hs + R (7), A/P + Hs + R (3)	—
	Porcelain	6.0 ± 3.0	A/D + Hs + R (5), Hs + R (3), A/P + Hs + R (4)	—

Super-Bond^b^	Metal^*^	9.2 ± 3.5	A/P + R (12)	—
	Composite	11.7 ± 2.1	R (2), A/P + R (10)	—
	Porcelain^*^	12.5 ± 2.2	R (2), A/P + R (10)	—

Single-Bond^c^	Metal	2.2 ± 1.2	A/D (8), A/P (2)	2
	Composite	1.3 ± 1.1	A/D (9)	3
	Porcelain	1.5 ± 1.0	A/D (9)	3

Variolink II^a^	Metal	2.0 ± 1.3	A/D (10)	2
	Composite	3.9 ± 4.0	A/D (10)	2
	Porcelain	5.0 ± 3.6	A/D (9), A/P + R (2)	1

^a,b,c^Significant differences in tensile strength between cements indicated by the different superscripts (*P* < 0.05).

^*^Differences in tensile strength between prostheses are significant.

A/D = adhesive failure at dentin side interface, A/P = adhesive failure at prosthesis side interface, R = cohesive failure in resin, Hs = cohesive failure in hybridized smear, and + = mixed failure.
